# Cost-Effectiveness Analysis of Six Strategies to Treat Recurrent *Clostridium difficile* Infection

**DOI:** 10.1371/journal.pone.0149521

**Published:** 2016-02-22

**Authors:** Lauren Lapointe-Shaw, Kim L. Tran, Peter C. Coyte, Rebecca L. Hancock-Howard, Jeff Powis, Susan M. Poutanen, Susy Hota

**Affiliations:** 1 Department of Medicine, University of Toronto, Toronto, Canada; 2 Department of Medicine, University Health Network, Toronto, Canada; 3 Institute of Health Policy, Management and Evaluation, University of Toronto, Toronto, Canada; 4 Department of Medicine, Toronto East General Hospital, Toronto, Canada; 5 Department of Medicine and Medical Microbiology, Mount Sinai Hospital, Toronto, Canada; Cleveland Clinic, UNITED STATES

## Abstract

**Objective:**

To assess the cost-effectiveness of six treatment strategies for patients diagnosed with recurrent *Clostridium difficile* infection (CDI) in Canada: 1. oral metronidazole; 2. oral vancomycin; 3.oral fidaxomicin; 4. fecal transplantation by enema; 5. fecal transplantation by nasogastric tube; and 6. fecal transplantation by colonoscopy.

**Perspective:**

Public insurer for all hospital and physician services.

**Setting:**

Ontario, Canada.

**Methods:**

A decision analytic model was used to model costs and lifetime health effects of each strategy for a typical patient experiencing up to three recurrences, over 18 weeks. Recurrence data and utilities were obtained from published sources. Cost data was obtained from published sources and hospitals in Toronto, Canada. The willingness-to-pay threshold was $50,000/QALY gained.

**Results:**

Fecal transplantation by colonoscopy dominated all other strategies in the base case, as it was less costly and more effective than all alternatives. After accounting for uncertainty in all model parameters, there was an 87% probability that fecal transplantation by colonoscopy was the most beneficial strategy. If colonoscopy was not available, fecal transplantation by enema was cost-effective at $1,708 per QALY gained, compared to metronidazole. In addition, fecal transplantation by enema was the preferred strategy if the probability of recurrence following this strategy was below 8.7%. If fecal transplantation by any means was unavailable, fidaxomicin was cost-effective at an additional cost of $25,968 per QALY gained, compared to metronidazole.

**Conclusion:**

Fecal transplantation by colonoscopy (or enema, if colonoscopy is unavailable) is cost-effective for treating recurrent CDI in Canada. Where fecal transplantation is not available, fidaxomicin is also cost-effective.

## Background

*Clostridium difficile* infection (CDI), a common healthcare-associated infection, is associated with significant morbidity and mortality.[1, 2] CDI results from exposure to *C*. *difficile* in the setting of disrupted intestinal microbiota, most commonly resulting from antibiotic use.[3] The standard treatment for a first episode of CDI is antibiotic therapy with either metronidazole or oral vancomycin.[4] Nonetheless, 15–28% of cases recur after discontinuation of antibiotics.[5–7] Patients who experience one recurrence are at a significantly higher risk of having additional recurrences.[8, 9]

The current standard of care for recurrent CDI is antibiotic therapy, with first recurrences treated with either metronidazole or oral vancomycin.[4] A prolonged taper-pulse course of oral vancomycin is recommended for all subsequent recurrences.[4] Recurrence rates with vancomycin vary widely in the literature, between 31% and 75%, with varying dose, duration of administration, as well as length of follow-up.[7, 9–12]

Fidaxomicin and fecal transplantation by various routes are alternatives that potentially offer lower rates of recurrence.[13, 14] However, both of these newer treatments have significant barriers to adoption. In the case of fidaxomicin, the high cost of the drug has been cited as a major disadvantage, one which has limited its coverage under provincial drug plans to one 10 day course.[15] The results of cost-effectiveness analyses of fidaxomicin for first episode and first recurrence have yielded conflicting results.[16, 17] A Canadian Cost-Benefit Analysis comparing fidaxomicin to vancomycin found that fidaxomicin cost an incremental $18,190 per second recurrence avoided.[18]

Similarly, fecal transplantation is a promising treatment for recurrent CDI, with cure rates reported between 80 and 94%.[9, 14] Despite low reported rates of adverse events with fecal transplantation, concerns about unknown health risks persist, including the risk of infectious disease transmission.[14] Consequently, Health Canada has restricted its use to clinical trials until recently.[19, 20] In addition to regulatory limitations, the cost of supporting processes, infrastructure and personnel are perceived as barriers to the adoption of fecal transplantation by clinicians and institutions.[21]

A cost-effectiveness analysis of treatments for recurrent CDI conducted using Medicare payment information from the United States found fecal transplantation by colonoscopy to be cost-effective compared to oral vancomycin alone for the treatment of recurrent CDI.[22] Conclusions from this study may not apply to other jurisdictions where systems and costs of care are different. In Canada, national and provincial policymakers (such as each province’s Ministry of Health) determine which treatment options are available to patients, and which will be publicly funded. The purpose of this study was to evaluate the cost-effectiveness of multiple treatment options for recurrent CDI, in order to inform Canadian policymakers, hospital managers and clinicians.

## Methods

We conducted a cost-effectiveness analysis of six treatment strategies for the first and subsequent recurrences of CDI.

### Treatments compared

1. 2-week course of oral metronidazole followed by a 6-week taper-pulse course of oral vancomycin for subsequent recurrences (this strategy will be referred to as “metronidazole”)
2. 2-week course of oral vancomycin followed by a 6-week taper-pulse course of oral vancomycin for subsequent recurrences (this strategy will be referred to as “vancomycin”)
3. 10-day course of oral fidaxomicin followed by a 6 week taper-pulse course of oral vancomycin for subsequent recurrences (this strategy will be referred to as “fidaxomicin”)
4. 2-week course of oral vancomycin with fecal transplantation via enema followed by the same (vancomycin and fecal transplantation by enema) using a different donor at each subsequent recurrence (this strategy will be referred to as “fecal transplantation by enema”)
5. 2-week course of oral vancomycin with fecal transplantation via nasogastric tube (NG) followed by the same (vancomycin and fecal transplantation by NG) using a different donor at each subsequent recurrence (this strategy will be referred to as “fecal transplantation by NG”)
6. 2-week course of oral vancomycin with fecal transplantation via colonoscopy followed by the same (vancomycin and fecal transplantation by colonoscopy) using a different donor at each subsequent recurrence (this strategy will be referred to as “fecal transplantation by colonoscopy”)

### Perspective

In Canada, the provincial Ministries of Health assume the cost of hospital care, physician services, and a varying proportion of outpatient drug costs. We adopted the perspective of the Ontario Ministry of Health and Long-Term Care for this study.

### Model Structure and Assumptions

**Fig 1:**
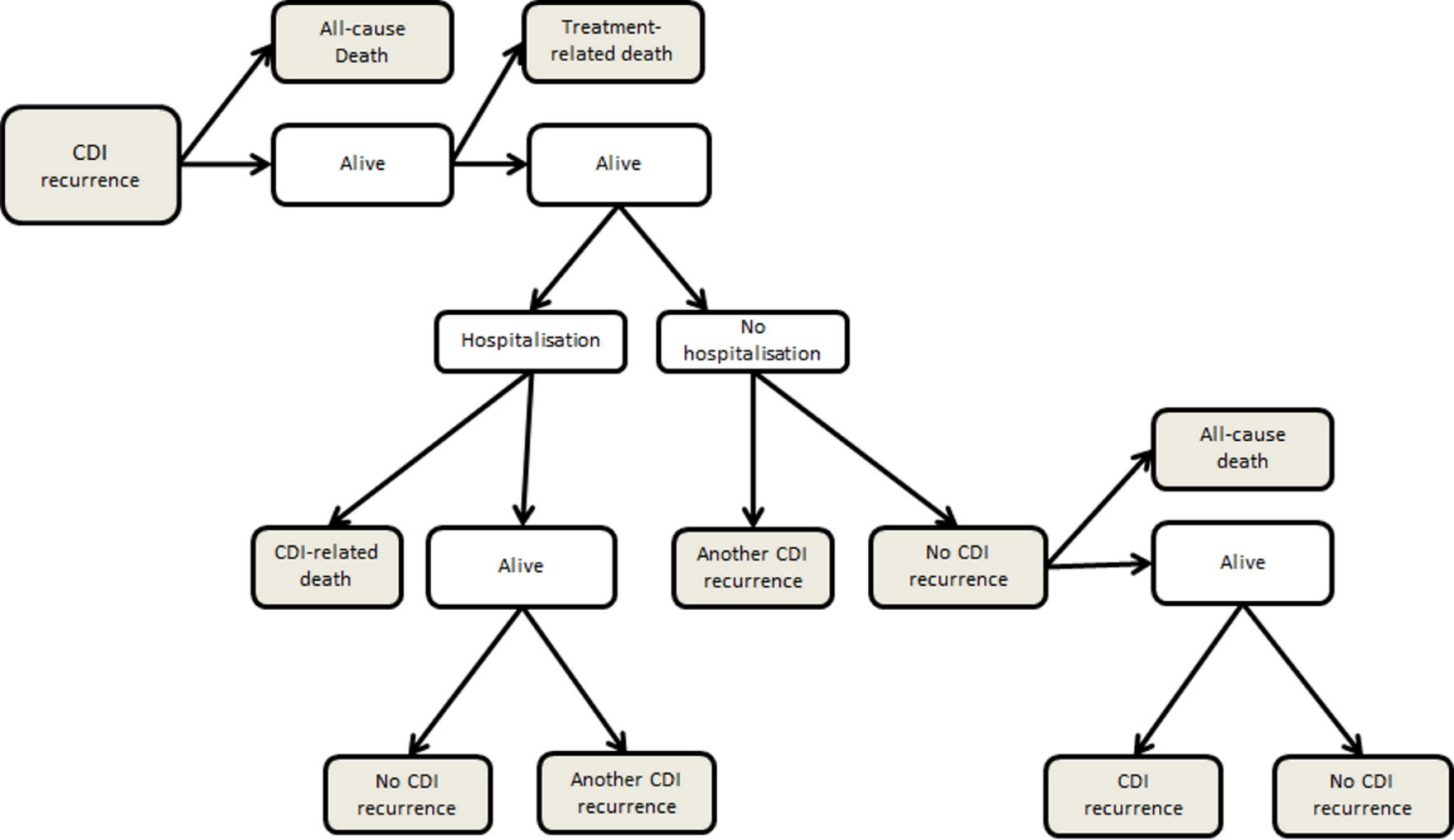
Clinical events modelled in each cycle. Markov states are shaded in grey. CDI = *Clostridium difficile* infection.

A decision-analytic model incorporating Markov processes was developed using TreeAge Pro 2013 (**S1 File**, TreeAge Software Inc., Williamstown, MA). The typical patient modelled in the study was a 70-year old community-dwelling person experiencing their first recurrence of CDI (the first episode of CDI was not included in the model). This represents the mean age of a patient with recurrent CDI in published studies.[9, 23] We assumed that recurrence and treatment could only occur once every six week cycle, reflecting the duration of the oral vancomycin taper-pulse, as well as the timing of recurrence, occurring within two weeks of antibiotic discontinuation in 81% of patients.[8] Thus, the modeled cycle length was six weeks. In the first cycle, all patients experienced a recurrence of CDI. In subsequent cycles, patients could be well (no recurrence), dead, or experience another recurrence, according to the events that occurred in the previous cycle (see Fig 1). As the mean number of CDI recurrences is just below three, we modelled up to three recurrences (three cycles) totaling an 18 week period.[8, 9] We did not model recurrences following 18 weeks because of the uncertain nature of the probability of recurrence over time.

Patients experiencing persistent diarrhea while being treated with oral metronidazole were deemed non-responders and were switched to oral vancomycin after six days of therapy, consistent with previous definitions of metronidazole non-response.[24] Patients with further recurrences after receiving metronidazole, fidaxomicin or vancomycin received a 6-week taper-pulse course of oral vancomycin.[4] Although current guidelines recommend considering fecal transplantation for a third recurrence following treatment with vancomycin, this option is not yet widely available in Canada, and patients are typically treated with repeat taper-pulse vancomycin. Dose and duration of all included antibiotics are detailed in Table 1 and are consistent with published guidelines.[4] Fidaxomicin dose and duration was consistent with that used in clinical trials.[13, 25]

**Table 1:** Medication doses and durations used in our analysis.

Agent	Dose	Frequency	Duration
Metronidazole	500 mg orally	Three times daily	14 days (6 days if no response)
Vancomycin*	125 mg orally	Four times daily	14 days
Vancomycin Taper-Pulse (6 weeks total)	125 mg orally four times daily for 14 days, followed by 125 mg orally twice daily for 7 days, followed by 125 mg orally once daily for 7 days, followed by 125 mg orally every other day for 7 days 125mg orally every third day for 7 days		
Fidaxomicin	200 mg orally	Twice daily	10 days

* 14-day course of vancomycin also used in fecal transplantation strategies.

Treatment with fecal transplantation by enema or nasogastric tube included a 14-day course of oral vancomycin to reflect practice in our centres, where donors are screened in real time. Donors are typically close friends or family identified by the patient; patients are treated with vancomycin while results of donor screening tests are pending. For fecal transplantation strategies, each treatment consisted of a single transplantation procedure.

### Model Outcomes

The primary outcomes were quality-adjusted life years (QALY) and costs in each treatment strategy. These were then used to compare strategies using the incremental cost-effectiveness ratio (ICER). QALYs are obtained by multiplying the time in a health state by the QALY weight of that health state, a measure of quality of life. A half-cycle correction was used for all QALYs in order to prevent systematic over- or under-estimation of payoffs with each cycle.[26] Treatment costs were assigned at the start of each cycle as this reflects when a diagnosis of recurrence and a treatment decision was made. Cost of hospitalization was modelled as a transitional cost in the middle of the cycle, for the proportion of recurrences requiring hospitalisation. We estimated the health effects of recurrences over the patient’s remaining lifetime, as is recommended by the Society of Medical Decision-Making.[27] This approach accounts for the years of life lost as a result of a CDI-related death and therefore could be expected to result in a lower ICER for treatments that reduce mortality relative to standard care. This was done by adding the QALY-weighted remaining life expectancy to the QALYs accrued in the final cycle of the model. The QALY weight used was that of an otherwise well community-dwelling person. Thus, the final cycle effectiveness for all non-death states was: 0.5*utility-weighted cycle length + discounted life expectancy*QALY weight.

### Model Parameters

Our source data were obtained from the published literature and hospital administrators; no unpublished patient-level information was used in this study.

#### Probabilities

Model transition probabilities were taken from published sources (see Table 2). Information on the proportion of outpatient cases of CDI admitted to hospital was obtained by multiplying the probability of hospitalization among CDI patients, 62% [28], by the probability that the primary reason for hospitalization was CDI, 28%.[29] This estimate for the probability of hospitalization was similar to the proportion of patients experiencing a non-recurrence complication of CDI from the Canadian Nosocomial Infection Surveillance Program (CNISP).[23] These complications included dehydration and gastrointestinal bleeding, conditions which would be expected to lead to hospital admission. Because criteria to define severe CDI are contradictory and have not been validated, and because our costing data and mortality estimates were for all hospitalized patients with CDI, we did not model severe CDI separately from CDI requiring hospitalization.[4, 30]

**Table 2:** Point estimates, range and distribution for all model variables.

Variable	Base Case	Range	Range type	Distribution Type	Standard Error
**Transition probabilities and relative risks**					
Probability of hospitalization for CDI [28, 29]	0.174	0.140–0.212	95% CI	Beta	0.018
Probability of response to oral metronidazole[31]	0.776	0.751–0.800	95% CI	Beta	0.012
Probability of recurrence after 2-week course of oral metronidazole[10]	0.4	0.053–0.853	95% CI	Beta	0.208
Probability of recurrence after 2-week course of oral vancomycin[9, 10, 25]	0.517	0.389–0.642	95% CI	Beta	0.066
Probability of recurrence after 6-week oral vancomycin taper-pulse[10]	0.178	0.059–0.431	95% CI	Beta	0.147
Relative risk of recurrence after 10-day course of fidaxomicin, compared to vancomycin[25]	0.620	0.360–1.07	95% CI	Log-normal	0.278
Probability of recurrence after fecal transplantation by enema[32]	0.185	0.063–0.381	95% CI	Beta	0.083
Probability of recurrence after fecal transplantation by nasogastric tube[9, 33–35]	0.233	0.155–0.334	95% CI	Beta	0.047
Probability of recurrence after fecal transplantation by colonoscopy[36–43]	0.078	0.050–0.119	95% CI	Beta	0.017
Relative risk of recurrence for additional 10 years of age[44]	1.16	1.07–1.26	95% CI	Log-normal	0.05
Probability of death from all causes, age 70[45, 46]	0.002	0.0015–0.0023	Range (men, women)	Beta	0.007
Probability of death from all causes, age 80[45, 46]	0.005	0.0043–0.0064	Range (men, women)	Beta	0.0005
Probability of death from colonoscopy[47]	0.0006	0.0003–0.0011	95% CI exact	Beta	0.0002
Probability of death from nasogastric tube insertion[48]	0.003	0.0003–0.0097	95% CI	Beta	0.002
Probability of death from CDI [23]	0.073	0.058–0.091	95% CI	Beta	0.008
Relative risk of death from CDI for additional ten years of age[49]	1.41	1.36–1.47	95% CI	Log-normal	0.03
**Medication Costs ($)**					
Metronidazole, 6 days	20	10–31	+/- 50%	Gamma	5
Metronidazole, 2-week course	39	19–58	+/- 50%	Gamma	10
Vancomycin, 2-week course	347	174–521	+/- 50%	Gamma	87
Vancomycin, 6-week taper-pulse	505	253–758	+/- 50%	Gamma	126
Fidaxomicin, 10-day course	2,405	1202–3607	+/- 50%	Gamma	601
**Costs of fecal transplantation by enema ($)**					
Day of procedure	174	87–261	+/-50%	Gamma	44
Outpatient visits	347	176–528	+/-50%*	Gamma	90
Laboratory testing	425	213–638	+/-50%	Gamma	108
Capital cost(equipment)	6844	47–6844	Range	Gamma	1,723
**Costs of fecal transplantation by nasogastric tube ($)**					
Day of procedure	226	113–339	+/-50%	Gamma	58
Outpatient visits	358	176–528	+/-50%*	Gamma	90
Laboratory testing	322	161–484	+/-50%	Gamma	82
Capital cost (equipment)	47	47–6844	**	Gamma	1,723
**Costs of fecal transplantation by colonoscopy ($)**					
Day of procedure[50]	588	294–882	+/-50%	Gamma	150
Outpatient visits	352	176–528	+/-50%	Gamma	90
Laboratory testing	374	187–561	+/-50%	Gamma	95
Capital cost (equipment)	3446	47–6844	**	Gamma	1,723
**Other costs**					
Hospitalization Cost[51]	16,800	8,266–65,512	-50%-95% UCL	Gamma,	24,853
Outpatient visits for patients treated with medications only	297	148–445	+/- 50%	Gamma	76
**QALY Weights**					
*Clostridium difficile* infection[52]	0.7	0.35–0.95	Plausible range	Beta	0.15
Community-dwelling 70–79 year old [53]	0.91	0.905–0.915	95% CI	Beta	0.0026
Community-dwelling 80+ year old [53]	0.88	0.87–0.89	95% CI	Beta	0.004
Dead	0	-	-	-	-
**Other**					
Number of patients eligible for fecal transplantation in institution-annual	79	40–119	+/- 50%	Gamma	19.75
Remaining life expectancy for a 70-year old (years)[45, 46]	16.41	15.13–17.68	Range	Normal	0.65
Remaining life expectancy for an 80-year old[45, 46]	9.75	8.93–10.57	Range	Normal	0.42

NG = nasogastric tube, CDI = *Clostridium difficile* infection.

* Ranges for cost of outpatient visits in fecal transplantation by NG and enema strategies are +/- 50% of the mean of the two.

** Range is extremes of capital cost for NG and enema protocols.

Our model did not distinguish between lack of clinical resolution and recurrence. We obtained the probability of recurrence after fecal transplantation from a systematic review.[54] We re-extracted source data from included studies to obtain estimates for upper gastrointestinal, enema and colonoscopy-delivered fecal transplantation. For fecal transplantation by NG, we updated the results of the systematic review to include a randomized controlled trial.[9] There is no published prospective data on recurrence rates after a 6-week vancomycin taper-pulse regimen. We obtained our estimate from a secondary analysis of a randomized controlled trial of patients with recurrent CDI; our estimate pooled together 19 patients receiving a tapering dose of vancomycin with 10 patients receiving a “taper-pulse” regimen.[10]

If confidence intervals and/or standard errors were not reported in source publications, exact binomial confidence intervals were calculated for individual studies. Where more than one study contributed to the point estimate, probabilities were pooled using a random effects model of pooled binomial proportions. These analyses were executed using the “binom” and “metaprop” packages available in R (R Foundation for Statistical Computing, Vienna, Austria, 2013).

#### Costs

All costs were reported in 2014 Canadian dollars. Costs obtained from previous years were inflated to 2014 value using the Consumer Price Index for Health and Personal Care.[55] In 2014, $1.00 in Canadian dollars was equivalent to $0.91 in United States dollars.[56]

Medication costs were obtained from a University Health Network (UHN) outpatient pharmacy (located at Toronto General Hospital) for a patient with Ontario Drug Benefit (ODB) coverage. The ODB (funded by the Ontario Ministry of Health and Long Term Care) covers the cost of drugs and the pharmacy mark-up fees for Ontario residents over the age of 65. [57] The costs reported represent what is paid by the ODB to dispensing pharmacies.

The cost of treatment with fecal transplantation included a single fecal transplantation procedure. Cost data for fecal transplantation by enema was provided by the investigators of a UHN clinical trial of fecal transplantation for the treatment of recurrent CDI.[58] Cost information for fecal transplantation by NG tube was provided by the Toronto East General Hospital (TEGH) according to their protocol. Laboratory, personnel, supply, and space costs were included. Table 3 outlines the components in the transplantation protocol for both sites, including which tests are done on stool donors and recipients. Laboratory and supply costs were obtained in 2011 for the enema protocol, and 2013 for the NG protocol.

**Table 3:** Components of fecal transplantation protocols. Costing for fecal transplantation by colonoscopy was derived from cost of fecal transplantation by enema and NG protocols (see methods). For this reason, there is no listed protocol for fecal transplantation by colonoscopy.

	Enema Protocol	NG Protocol
**Recipient baseline tests**		
Stool culture for enteric pathogens	X	
Stool ova and parasites	X	
Stool *Helicobacter pylori* antigen assay	X	
Storage of blood samples for testing if seroconversion	X	
**Donor screening tests**		
Stool culture for enteric pathogens	X	X
Stool for ova and parasites	X	X
Stool for *Clostridium difficile* toxin	X	X
Stool culture for methicillin-resistant *Staphylococcus aureus*	X	
Stool culture for vancomycin-resistant enterococci	X	
*Helicobacter pylori* stool antigen assay	X	
*Helicobacter pylori* serology assay	X	X
Hepatitis A, B and C serology	X	X
HTLV 1/2 serology	X	X
HIV test	X	X
Syphilis screen	X	X
**Donor fecal transplantation preparation**		
Stool preparation	X	X
**Personnel**		
Physician	X	X
Nurse	X	X
Lab technician	X	X
Radiology technician		X
**Clinic**		
Medical day unit	X	
Outpatient clinic	X	X
**Capital investment**		
Stomacher 400 Circulator (Seward, UK), Bag rack and Bags	X	
Blender		X

Personnel costs were obtained using hourly wage data for registered nurses from the Toronto region, as reported by Statistics Canada, with 13% added to account for benefits, as recommended by the Ontario Nursing Association.[59, 60] Physician fees were determined using the Ontario Schedule of Benefits.[57] Counselling and consent, which was performed by physicians, are included in these fees. Space costs were provided by the accounting department or department managers and include overhead (space, maintenance) costs.

The cost of a colonoscopy procedure (without physician fees) was obtained from a microcosting study from the neighbouring province of Quebec, in Canada.[50] Physician fees for a full colonoscopy were added to this, according to the Ontario Schedule of Benefits.[57] Cost of nursing time post-procedure and processing of stool were added to this, based on the mean of these costs for the NG and enema protocols. The cost of laboratory testing with the fecal transplantation by colonoscopy strategy was similarly obtained from the mean of these costs in the NG and enema strategies.

Capital costs for equipment used to prepare stool were included in the fecal transplantation strategies, and are listed separately. In the enema protocol this was a Stomacher 400 Circulator (Seward, UK), whereas in the NG protocol this was a typical household blender. The capital cost for stool preparation equipment in fecal transplantation by colonoscopy was taken as the mid-point of these two figures. Capital costs were annuitized using a 5% discount rate over five years. The annual cost was then distributed over the number of CDI cases seen annually at UHN to derive the typical cost of use per treatment. The cost of a colonoscope was included in the per-procedure cost of colonoscopy, as reported by Sharara et al.[50]

The cost of two outpatient visits was included in each treatment strategy. In addition, the fecal transplantations strategies included an outpatient visit for the stool donor. Costs of visits for donors and recipients in fecal transplantation strategies were obtained from UHN and TEGH. The mean cost from these two sources was used to provide a cost for outpatient visits in the fecal transplantation by colonoscopy strategy. In order to make an appropriate comparison with the fecal transplantation strategies, the cost of two outpatient visits was added to the medication-only treatment strategies. This was obtained from the mean cost of outpatient visits for fecal transplantation recipients in the fecal transplantation strategies.

Hospital Admission Costs, including the cost of in-hospital medications to treat CDI, were obtained from the Ontario Case Costing Initiative.[51] We extracted cost data for all patients over age 70 admitted in 2010–2011 with a most responsible diagnosis of A047 “Enterocolitis due to *Clostridium difficile*”.

#### Utilities and QALY weights

Utility measures for CDI have not been established through commonly accepted techniques. A previous cost-effectiveness analysis of CDI used utility measures for grade 3 and 4 chemotherapy-associated diarrhea to estimate the utility of CDI.[61] This value (0.3) is much lower than the utilities used for other gastrointestinal conditions, such as colitis (0.7) and irritable bowel syndrome (0.675).[52, 62] With the aim of being conservative, we used 0.7 as the utility for CDI; however we varied this utility widely in our sensitivity analysis.

We used a QALY weight for the well state obtained from a Health Utilities Index survey of community dwelling Canadians over age 70.[53] QALYs accrued by each strategy were obtained by multiplying the QALY weight of a state by the time spent in that state. A discounting rate of 5% was applied to QALYs over the patient’s remaining lifetime, consistent with recommendations from the Canadian Agency for Drugs and Technology in Health.[63]

### Analysis

All analyses of cost-effectiveness were made using a willingness-to-pay of $50,000/QALY. Following analysis of the base-case, one-way sensitivity analysis was carried out on all variables within their ranges. In addition, we tested our model using a 0% discount rate for lifetime QALYs.

Variables found to have the greatest impact on base-case results were further analyzed in a two-way sensitivity analysis at a willingness-to-pay of $50,000/QALY. A probabilistic sensitivity analysis (PSA), using 10,000 Monte Carlo cohort-based simulations, was used to simultaneously assess the effect of uncertainty in all parameters on model conclusions.

We also conducted several scenario analyses, to examine how model conclusions were altered by patient age, fidaxomicin patent status, access to fecal transplantation procedures, and the number of recurrences. The cost of generic fidaxomicin is expected to be 25% of the per-unit cost of the brand-name drug.[64]

## Results

### Base-case analysis

Fecal transplantation by colonoscopy was dominant in the base case, as it was cost-saving and more effective than all other treatment options. This strategy led to $140 saved and 0.31 additional QALYs compared to treatment with metronidazole. Per 1,000 patients treated, fecal transplantation by colonoscopy resulted in 439 fewer recurrences, 76 avoided hospitalizations, and 31 lives saved, compared to metronidazole (see Table 4). Vancomycin, fidaxomicin, fecal transplantation by NG and fecal transplantation by enema were also more expensive and less effective than fecal transplantation by colonoscopy (see Table 5).

**Table 4:** Health outcomes of each treatment strategy, per 1,000 patient cohort.

Strategy	Count of recurrences after the first	Count of hospitalisations	Count of CDI-related deaths*	Average life years
Vancomycin	636	284	119	14.46
Metronidazole	583	275	115	14.78
Fecal transplantation by NG	426	247	108	14.87
Fidaxomicin	458	253	106	14.90
Fecal transplantation by enema	340	233	98	15.04
Fecal transplantation by colonoscopy	144	199	84	15.26

* includes deaths from treatment-related complications.

**Table 5:** Cost and QALY accrued with each treatment strategy.

Treatment	Cost (2014 Canadian dollars)	QALY	ICER
**Scenario 1: base case**			
Fecal transplantation by colonoscopy	5,246	9.40	Dominates
Vancomycin	5,929	9.03	(Dominated)
Metronidazole	5,386	9.09	(Dominated)
Fecal transplantation by NG	5,935	9.15	(Dominated)
Fidaxomicin	7,319	9.16	(Dominated)
Fecal transplantation by enema	5,667	9.26	(Dominated)
**Scenario 2: patient is ten years older**			
Fecal transplantation by colonoscopy	5,310	6.02	Dominates
Vancomycin	6,174	5.63	(Dominated)
Metronidazole	5,598	5.69	(Dominated)
Fecal transplantation by NG	6,116	5.77	(Dominated)
Fidaxomicin	7,494	5.77	(Dominated)
Fecal transplantation by enema	5,815	5.87	(Dominated)
**Scenario 3: fidaxomicin is off-patent**			
Fecal transplantation by colonoscopy	5,246	9.40	Dominates
Vancomycin	5,929	9.03	(Dominated)
Metronidazole	5,386	9.09	(Dominated)
Fecal transplantation by NG	5,935	9.15	(Dominated)
Fidaxomicin	5,521	9.16	(Dominated)
Fecal transplantation by enema	5,667	9.26	(Dominated)
**Scenario 4: no fecal transplantation option available**			
Metronidazole	5,386	9.09	
Fidaxomicin	7,319	9.16	25,968
Vancomycin	5,929	9.03	(Dominated)
**Scenario 5: no colonoscopy available**			
Metronidazole	5,386	9.09	
Fecal transplantation by enema	5,667	9.26	1,708
Vancomycin	5,929	9.03	(Dominated)
Fecal transplantation by NG	5,935	9.15	(Dominated)
Fidaxomicin	7,319	9.16	(Dominated)
**Scenario 6: two cycles only (single recurrence after the first)**			
Metronidazole	4,793	9.14	
Fecal transplantation by colonoscopy	4,918	9.38	514
Vancomycin	5,341	9.07	(Dominated)
Fidaxomicin	6,722	9.21	(Dominated)
Fecal transplantation by NG	5,058	9.24	(Dominated)
Fecal transplantation by enema	4,954	9.31	(Dominated)

### Sensitivity Analyses

**Fig 2:**
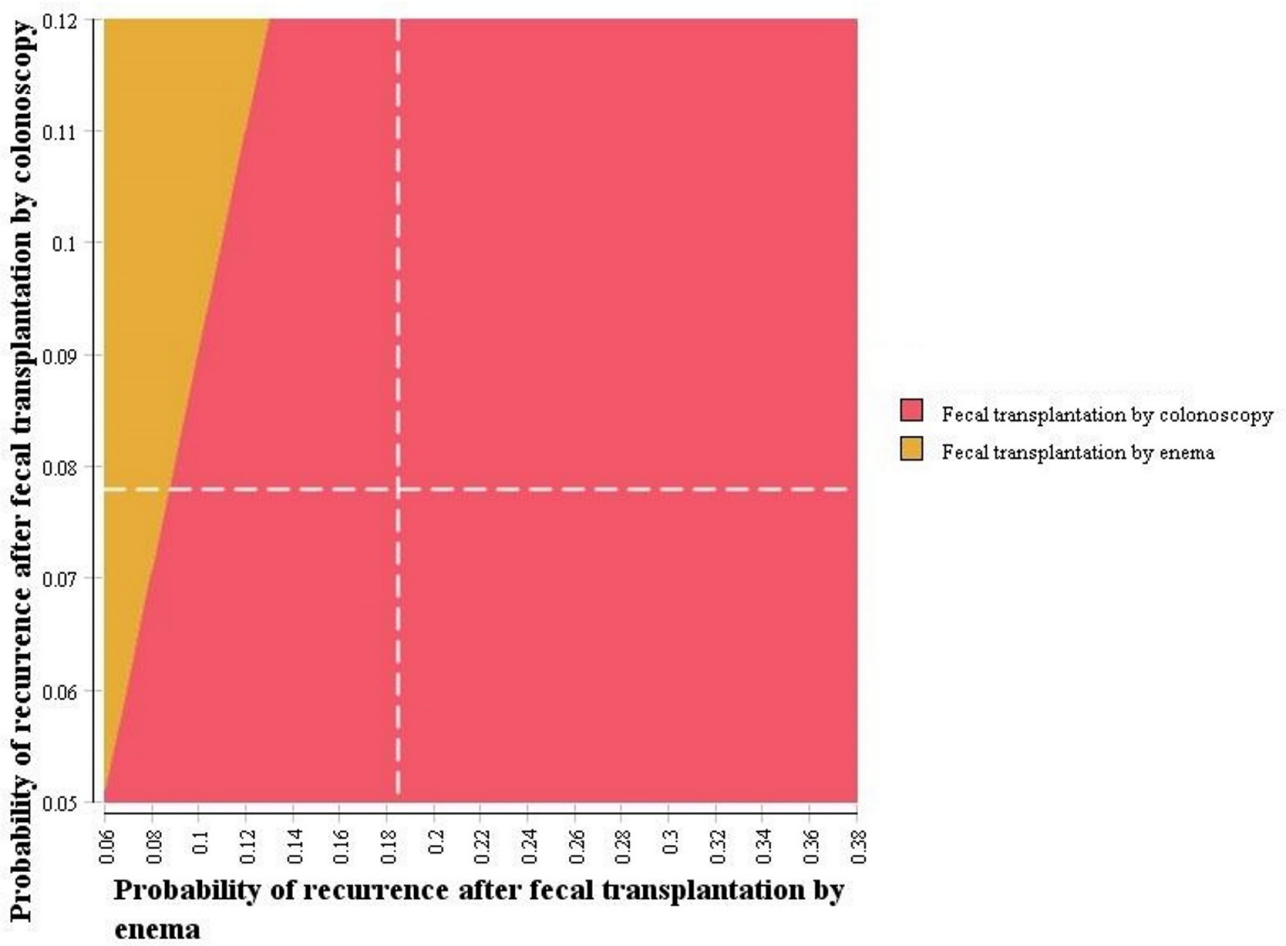
Two-way sensitivity analysis for the preferred strategy according to probability of recurrence following fecal transplantation by colonoscopy or enema. The colour indicates which strategy leads to the greatest number of QALYs at a willingness-to-pay of $50,000/QALY. The hatched white lines identify the values used in the base case. $CAN = Canadian dollars.

Varying all parameters within their stated ranges did not change the preferred treatment strategy, with one exception. Fecal transplantation by enema became the preferred strategy when the probability of recurrence following this strategy dropped below 8.7%. The effect of uncertainty in the probability of recurrence following fecal transplantation by colonoscopy and enema strategies is explored in Fig 2.

Varying costs within their stated ranges did not change the preferred strategy. The total treatment costs (capital and non-capital) for fecal transplantation by colonoscopy would have to exceed $8,062 per treatment before fecal transplantation by enema became the preferred strategy. Further, as long as the total per-treatment costs were below $1,446, fecal transplantation by colonoscopy was cost-saving compared to all alternative strategies. Removing the discount rate for future QALYs did not change model conclusions: fecal transplantation by colonoscopy remained dominant over all other options.

**Fig 3:**
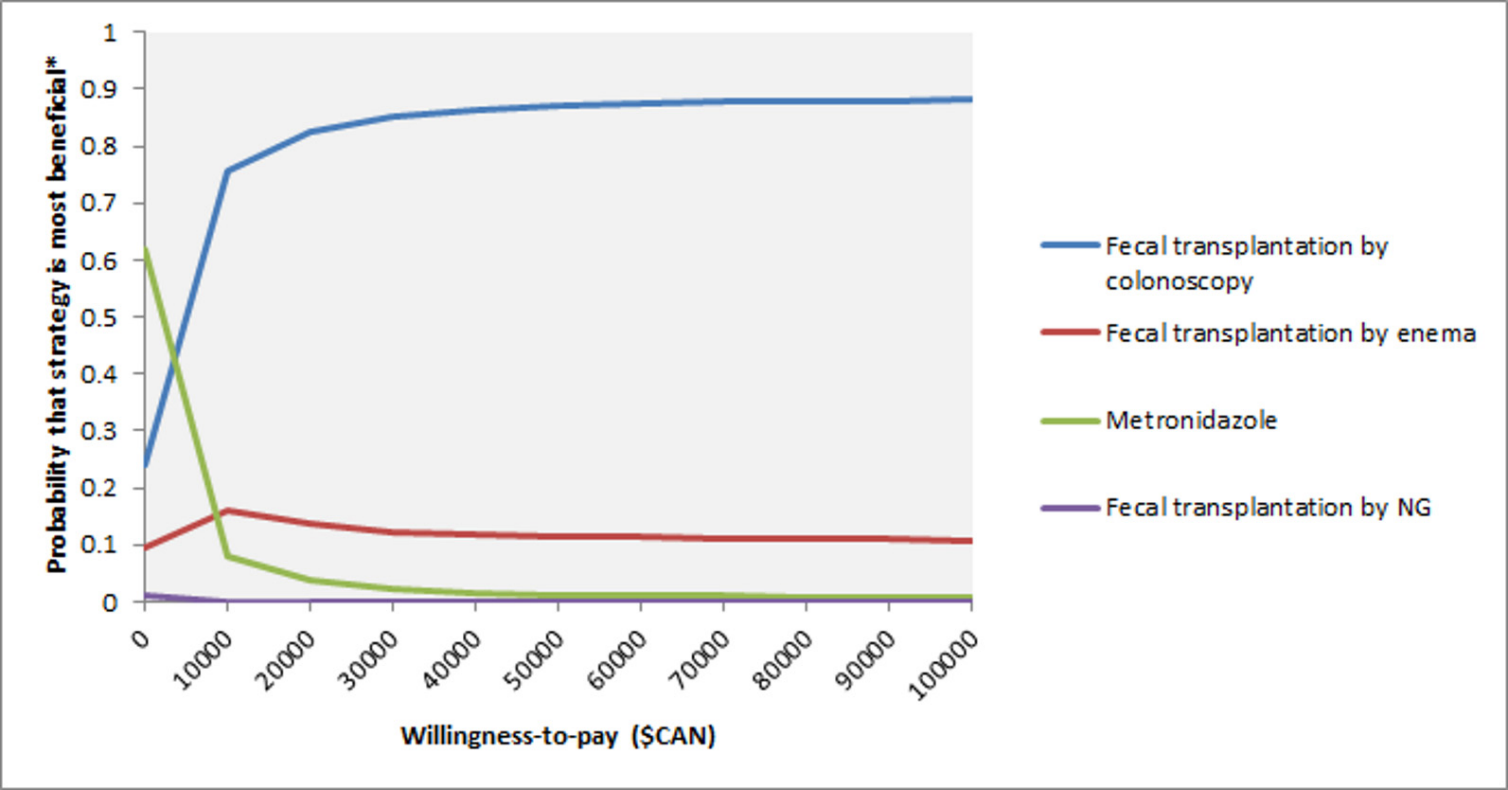
Cost-effectiveness acceptability curve. *the most beneficial strategy is the one that leads to the greatest number of QALYs at each willingness-to-pay threshold. Vancomycin and fidaxomicin strategies are not included in this figure as the probability that either strategy was most beneficial was 0 at all included thresholds. $CAN = Canadian dollars. NG = nasogastric tube.

Probabilistic sensitivity analysis with 10,000 Monte Carlo trials demonstrated that fecal transplantation by colonoscopy was the most beneficial strategy in 87% of trials at a willingness-to-pay of $50,000/QALY.(Fig 3).

### Scenario Analyses

As a result of the higher rate of CDI recurrence and CDI-related mortality in older patients, fecal transplantation by colonoscopy was even more advantageous in this population ($288 saved and 0.33 additional QALYs compared to metronidazole). Fidaxomicin coming off-patent, while it would reduce the average cost of this strategy by 23%, did not change the preferred strategy, which remained fecal transplantation by colonoscopy (see Table 5).

In a setting with no access to colonoscopy, fecal transplantation by enema was cost-effective, with an ICER of $1,708/QALY gained compared to the metronidazole strategy. Meanwhile, if fecal transplantation in any form is not available, fidaxomicin became cost-effective, with an ICER of $25,968/QALY gained compared to metronidazole. Finally, running the model for only two cycles (one recurrence after the first) identifies fecal transplantation by colonoscopy as a cost-effective alternative to metronidazole (see Table 5).

## Discussion

In our cost-effectiveness analysis of treatments for recurrent CDI, fecal transplantation by colonoscopy was dominant in the base case, as it was both more effective and less costly than all other options. Our results were sensitive to uncertainty in the probability of recurrence with fecal transplantation by enema. Where the probability of CDI recurrence with fecal transplantation by enema was below 8.7%, it became the preferred strategy. After accounting for uncertainty in all parameters, there was an 87% probability that fecal transplantation by colonoscopy was the most beneficial strategy at our willingness-to-pay threshold. Where colonoscopy or fecal transplantation by any means was not available, fecal transplantation by enema and fidaxomicin were, respectively, cost-effective treatment strategies.

Numerous observational studies, and one randomized controlled trial, have associated fecal transplantation for recurrent CDI with low rates of recurrence.[9, 14] Until recently, access to fecal transplantation in Canada has been limited to study settings. With the relaxation of Health Canada regulations[20], the lack of facilities offering such treatments may now become the primary barrier to treatment. This could result from an absence of institutional support, leading to a lack of resources necessary for delivery. Our study provides evidence for hospital leaders and healthcare funders that providing fecal transplantation is worthwhile. Where colonoscopy is not available, fecal transplantation by enema can be adopted with little up-front expense if a household-style blender is used for stool preparation, rather than a Stomacher.

Our conclusions are limited by the quality of our parameter estimates. Probabilities for recurrence following vancomycin taper-pulse and fecal transplantations were obtained almost entirely from observational studies, most of which were deemed to be of intermediate or low quality according to the National Institute for Health and Care Excellence checklist for quality of case series data.[14] The little randomized controlled trial evidence available came from a very small trial, with only 16 participants.[9] Data on fidaxomicin, although originating from trials, is limited by short follow-up periods (4 weeks), possibly underestimating recurrence in both fidaxomicin and oral vancomycin arms.[25] Estimates of relative cost-effectiveness will require revision if the results of future randomized controlled trials differ from existing estimates.

Costs of treatment with each modality will vary across jurisdictions, resulting in different conclusions and/or varying ICERs. For instance, costs of similar processes, used for fecal transplantation by NG or enema, varied between institutions within the same city. Capital costs for equipment used for stool preparation were particularly variable in our study. Further, the capital cost for fecal transplantation by colonoscopy did not include the cost of the colonoscope itself, since this was included in the per-procedure cost reported by Sharara et al.[50] Their estimates were obtained in a setting with a large volume of colonoscopies performed (greater than 4,000 colonoscopy procedures per year). In a lower-volume setting, the capital cost of acquiring a colonoscope for the purpose of treating a relatively small number of patients with recurrent CDI may be considered prohibitive. However, our results indicate that even at a high per-procedure cost, fecal transplantation by colonoscopy is cost-effective.

In our analysis we assumed that probability of recurrence remained fixed over time, yet in fact recurrence risk probably declines over time if a first recurrence has not occurred.[65] Conversely, risk of recurrence is thought to rise in relation to the number of recurrences already experienced.[12] As such, the data on vancomycin dose, duration and recurrence is likely confounded by the number of previous recurrences. In the absence of large prospective cohort or trial data for patients experiencing their first recurrence of CDI, generating probabilities specific to the number of recurrences would be challenging.

We did not model colectomy as a distinct state. Colectomy is rare, occurring in 1% or less of cases of CDI over the age of 65, and associated with a high mortality.[66–68] The inclusion of colectomy, a rare yet expensive complication, can be expected to increase the difference between groups, and make all treatments with improved efficacy appear more cost-effective, thus not altering the direction of our model conclusions.

We did not model adverse drug events because of the mild and transient nature of reported events. Both oral vancomycin and fidaxomicin have minimal systemic absorption, and reported adverse events were mild and transient, consisting of symptoms that could also be effects of CDI (most common: nausea, vomiting, fever, hypokalemia).[13] Our model accounted for the small mortality risk resulting from NG insertion and colonoscopy procedures, but dit not include any variable for risk of exposure to fecal transplantation material itself. In a systematic review, only 3 of 11 studies of fecal transplantation reported any adverse events, for an event rate of three possible events for 273 treated patients; in these patients, described complications (upper gastrointestinal bleed, peritonitis and enteritis) may have related to the use of an NG tube rather than the stool material itself.[14]

### Comparison with other work

A cost-effectiveness analysis of strategies for treatment of recurrent CDI using U.S. data concluded that fecal transplantation by colonoscopy was cost-effective with an ICER of $17,016 compared to vancomycin.[22] Unlike these authors, we adopted a lifetime perspective over which to model QALYs, as is recommended by the International Society of Pharmacoeconomics and Outcomes Research.[27] This approach accounts for the quality-adjusted years of life lost as a result of a CDI-related death. Cost data in our study was obtained using real hospital costs in a single payer publicly funded healthcare system with universal coverage, rather than reimbursements by Medicare, which insures only a portion of the population. In our study, fecal transplantation by colonoscopy was cost-saving, as a result of fewer hospitalisations occurring in this treatment group.[22] As the cost of hospitalisation used in our study was greater than that used by Konijeti et. al, this could explain the difference in results.

## Conclusions

Recurrent CDI is associated with significant morbidity and mortality, however several treatment options are available. Our cost-effectiveness analysis revealed that fecal transplantation by colonoscopy is cost-effective for the treatment of recurrent CDI. In a setting where colonoscopy is not available, fecal transplantation by enema is a cost-effective alternative. Our conclusions are limited by the quality of data on the effectiveness of fecal transplantation and taper-pulse vancomycin. The availability of fecal transplantation is dependent on institutional support for the procedure. Healthcare leaders should note that although there may be upfront costs related to a fecal transplantation program, providing such a service for patients with recurrent CDI is likely worth the cost.

## Supporting Information

S1 File Treeage file used to model base case results. (TREX)
